# Volumetric medical image compression using 3D listless embedded block partitioning

**DOI:** 10.1186/s40064-016-3784-y

**Published:** 2016-12-20

**Authors:** Ranjan K. Senapati, P. M. K Prasad, Gandharba Swain, T. N. Shankar

**Affiliations:** 1 Department of ECE, K L University, Vaddeswaram, Guntur, Andhra Pradesh 522502 India; 2 GMR Institute of Technology, Rajam, Srikakulam, 532127 India; 3 Department of CSE, K L University, Vaddeswaram, Guntur, Andhra Pradesh 522502 India

**Keywords:** 3D hierarchical listless embedded block, Set partitioning in hierarchical trees, Volumetric compression, Embedded coder, Peak-signal-to-noise-ratio

## Abstract

This paper presents a listless variant of a modified three-dimensional (3D)-block coding algorithm suitable for medical image compression. A higher degree of correlation is achieved by using a 3D hybrid transform. The 3D hybrid transform is performed by a wavelet transform in the spatial dimension and a Karhunen–Loueve transform in the spectral dimension. The 3D transformed coefficients are arranged in a one-dimensional (1D) fashion, as in the hierarchical nature of the wavelet-coefficient distribution strategy. A novel listless block coding algorithm is applied to the mapped 1D coefficients which encode in an ordered-bit-plane fashion. The algorithm originates from the most significant bit plane and terminates at the least significant bit plane to generate an embedded bit stream, as in 3D-SPIHT. The proposed algorithm is called 3D hierarchical listless block (3D-HLCK), which exhibits better compression performance than that exhibited by 3D-SPIHT. Further, it is highly competitive with some of the state-of-the-art 3D wavelet coders for a wide range of bit rates for magnetic resonance, digital imaging and communication in medicine and angiogram images. 3D-HLCK provides rate and resolution scalability similar to those provided by 3D-SPIHT and 3D-SPECK. In addition, a significant memory reduction is achieved owing to the listless nature of 3D-HLCK.

## Background

As the amount of patient data increases, compression techniques for the digital storage and transmission of medical images become mandatory. Imaging modalities such as ultrasonography (US), computer tomography (CT), magnetic resonance imaging (MRI) and X-rays provide flexible means of viewing anatomical cross sections for diagnosis. Three dimensional (3D) medical images can be viewed as a time sequence of radiographic images, the tomographic slices (images) of a dynamic object, or a volume of a tomographic slice images of a static object (Udupa and Herman 2000). In this paper, a 3D medical image corresponds to a volume of tomographic slices, which is a rectangular array of voxels with certain intensity values. Progressive lossy to lossless compression from a unified bit string is highly desirable in medical imaging. Lossy compression is tolerated as long as the required diagnostic quality is preserved. Lossless to lossy compression techniques are primarily used in telemedicine, teleradiology and the wireless monitoring of capsule endoscopy.

A compression technique using wavelets provides better image quality compared to joint photographic experts group compression (JPEG) (Pennebaker and Mitchell 1993; Santa-cruz et al. 2000). It also provides a rich set of features such as a progressive in quality and resolution, the region of interest (ROI) and optimal rate-distortion performance with a modest increase in computational complexity. The JPEG standard uses an 8 × 8 discrete cosine transform (DCT) and the JPEG2000 standard uses two dimensional discrete wavelet transform (2D-DWT). The Karhunen–Loueve transform (KLT) is an optimal method for encoding images in the mean squared error (MSE) sense. The compression performance of 2D cosine, Fourier, and Hartley transforms was compared using positron emission tomography (PET) and magnetic resonance (MR) images in Shyam Sunder et al. (2006). The authors claimed that the discrete Hartley transform (DHT) and the discrete Fourier transform (DFT) perform better than the DCT. Several techniques based on the three-dimensional discrete cosine transform (3D-DCT) have been proposed for volumetric data coding (Tai et al. 2000). Nevertheless, these techniques fail to provide lossless coding coupled with quality and resolution scalability, which is a significant drawback for teleradiology and telemedicine applications.

Several works on wavelet-based 3D medical image compression have been reported in the literature (Schelkens et al. 2003; Xiong et al. 2003; Chao et al. 2003; Gibson et al. 2004; Xiaolin and Tang 2005; Sriram and Shyamsunder 2011; Ramakrishnan and Sriram 2006; Srikanth and Ramakrishnan 2005; He et al. 2003). A method based on block-based quad-tree compression, layered zero-coding, and context-based arithmetic coding was proposed by Schelkens et al. (2003). They claimed that the method gives an excellent result for lossless compression and a comparable result for lossy compression. Modified 3D-SPIHT and 3D-EBCOT schemes for the compression of medical data were proposed by Xiong et al. (2003). Their method gives a comparable result for lossy and lossless compression. An optimal 3D coefficient tree structure for 3D zero-tree coding was proposed by Chao et al. (2003). They suggested that an asymmetrical tree can produce a higher compression ratio than a symmetrical one. Gibson et al. (2004) incorporated an ROI and texture modelling stage into the 3D-SPIHT coder for the compression of angiogram video sequences based on bit allocation criteria. Xiaolin and Tang (2005) presented a 3D scalable coding scheme which aimed to improve the productivity of a radiologist by providing a high decoder throughput, random access to the coded data volume, progressive transmission, and coding gain in a balanced design approach. Sriram and Shyamsunder (2011) proposed an optimal coder by making use of wavelets db4, db6, cdf9/7, and cdf5/3 with 3D-SPIHT, 3D-SPECK, and 3D-BISK. They found that cdf 9/7 with 3D-SPIHT yields the best compression performance. Ramakrishnan and Sriram (2006) proposed a wavelet-based SPIHT coder for DICOM images for teleradiology applications. Similarly, many works based on 3D-SPECK, 3D-BISK, and 3D-SPIHT used for the compression of hyperspectral images have been reported (Tang et al. 2003; Fowler and Rucker 2007; Lu and Pearlman 2001).

3D-SPIHT and 3D-SPECK use auxiliary lists [e.g., a list of insignificant pixels (LIP), a list of insignificant sets (LIS), and a list of significant pixels (LSP)] for tree/block partitioning. The auxiliary lists demand an efficient memory management technique, as the coefficients in the list are shuffled out during bit-plane partitioning. This feature is not favorable for hardware realisation. Therefore, 2D variants of listless coders called no list SPIHT (NLS) (Latte et al. 2006), listless SPECK (Wheeler and Pearlman 2000), LEBP (Senapati et al. 2013), and HLDTT (Senapati et al. 2014a) use a state table to keep track of set partitions. These listless coders can be efficiently realised in hardware. Recently, a listless implementation of 3D-SPECK for the compression of hyperspectral images was proposed by Ngadiran et al. (2010).

To the best of the authors’ knowledge, there have been few works on 3D listless implementations for medical images in the literature. This motivates us to develop a novel technique for encoding medical images using a modified 3D listless technique. The 3D listless algorithm uses static and dynamic marker state tables for encoding large clusters of insignificant blocks, which results in a rate reduction at earlier passes. From a unified bit string, the algorithm provides rate and resolution scalability for the compression of volumetric data. This set of features is a potential requirement in telemedicine and teleradiology applications.

The organization of the paper is as follows: “The proposed 3D-HLCK embedded coder” section presents the proposed 3D-HLCK algorithm and its memory allocation for 3D medical images. Simulation result and analysis with respect to coding performances and computational complexity using big-O notation are presented in “Results and discussion” section. Conclusions and further research directions are provided in “Conclusion” section.

## The proposed 3D-HLCK embedded coder

**Fig. 1:**
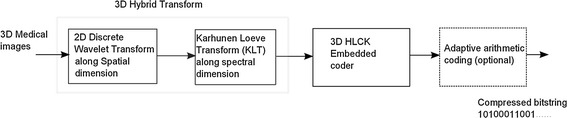
Proposed 3D hierarchical listless embedded coder

**Fig. 2:**
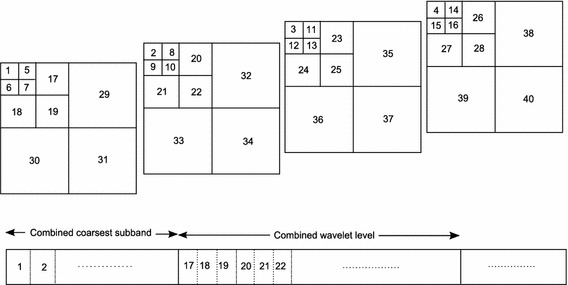
Scanning pattern of the subbands in medical MRI images and mapping to 1D array

The block diagram of the proposed 3D-HLCK algorithm is shown in Fig. 1. The 3D hybrid transformation is carried out in the 1st stage. Then, all 3D coefficients are mapped to one dimensional for processing by the proposed 3D-HLCK algorithm. Figure 2 shows the coefficient arrangement algorithm. The arrangement is created by keeping in mind the hierarchical nature of a wavelet pyramid. Four image slices are shown here as an illustration. The experiment is carried out for eight slices in all images. The coefficients in each slice undergo Z-scanning which maps two dimension to one dimension.

**Fig. 3:**
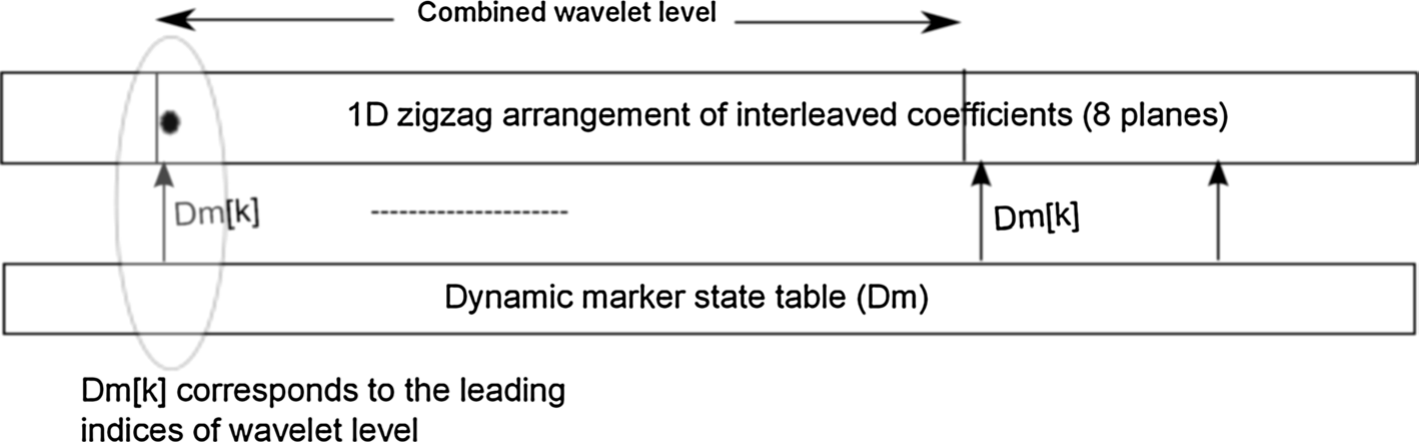
The association between *Dm*[*k*] values and coefficient values $\xi (k)$, where $k=0,1,2,\ldots$

The coefficient is accessed using a linear indexing scheme (Wheeler and Pearlman 2000). Two types of marker state tables are used. They are a (1) dynamic marker table (**Dm**) and (2) static marker table (**Sm**). The one-to-one correspondence between the coefficient values and the marker values are shown in Fig. 3. All marker values are initialised and loaded into memory along with the one dimensional (1D) arrangement of the image coefficient values. The dynamic markers in **Dm** update the values to indicate partitioning. The partitioning can be octal (8), tri (3) or quad (4). Octal partitioning takes place while there is a search for the significant coefficient in a composite wavelet level. Tri partitioning takes place while there is a search for the significant coefficient in a wavelet level. Quad partitioning takes place if a coefficient is found to be significant in a wavelet subband or a subblock inside a subband. The static marker table **Sm** is only used to skip a large cluster of areas, e.g. the entire composite level/wavelet level/wavelet subband. The length of the dynamic marker table is the same as that of the image array length. If each marker in the dynamic marker state table is 4 bits, then the memory consumes *I*/2 bytes for the state table. There are only three fixed markers per wavelet level. For five levels, there will be 15 × 3 = 45 markers in the constant marker table. The values of the markers depend on the image size (i.e. *N* × *N*) and the level of wavelet decompositions *L*. The initial marker value is $(log_2N-L+1)$, and the final value is $(log_2N+1)$. For example, if the image dimension *N* = 128 and the level of decomposition *L* = 5, the marker values are 3, 4, 5, 6, 7, and 8 in each leading node of the wavelet level. Each bit plane undergoes three passes, as in conventional 3D-SPIHT. They are (1) an insignificant coefficient pass, (2) an insignificant set pass, and (3) a refinement pass.

During the insignificant coefficient pass, a single coefficient will be tested for significance. During the insignificant set pass, a composite level/individual level/individual subband will be tested for significance. The refinement pass successively reduces the uncertainty interval between the reconstructed coefficient value and the actual coefficient value.

The symbol and meaning of each type of marker are specified below

- INC: The coefficient is insignificant or untested for this bit plane.
- NSC: The coefficient becomes significant so it shall not be refined for this bit plane.
- SCR: The coefficient is significant and it shall be refined in this bit plane.

The markers listed below corresponds to the leading indices of each lower level of the pyramid. These markers shall be used to test the insignificance of a subband/block during each bit-plane pass.

Static markers (*Sm*[*k*]):

- *Sm*[1]: The coefficient is at the leading index of the combined wavelet level *L*. All the coefficients in the same wavelet level shall be skipped.
- *Sm*[129]: The coefficient is at the leading index of the combined wavelet level *L* − 1. All coefficients in the same wavelet level shall be skipped.
- *Sm*[513]: The coefficient is at the leading index of the combined wavelet level *L* − 2. All coefficients in the same wavelet level shall be skipped.
$$\vdots$$
- *Sm*[32,719]: This coefficient is at the leading index of the finest pyramid level $L-5$. All coefficients in this level shall be skipped.

Dynamic markers (*Dm*[*k*]): The partitioning take place due to dynamic markers in a typical pyramid level (L-1) is illustrated below. Similar illustration can be applied for other levels.

- If *Dm*[129] = *Sm*[129], then the combined wavelet level *L* − 1 may be skipped.
- If *Dm*[129] = *Sm*[129]-1, then a wavelet level (for a single plane) *L* − 1 may be skipped.
- If *Dm*[129] = *Sm*[129]-2, then a single subband block in the wavelet level *L* − 1 may be skipped.
- If *Dm*[129] = *Sm*[129]-3, then $\frac{1}{4}$th of a subband block from a wavelet level may be skipped.
$$\vdots$$
- If *Dm*[129] = 0, then a single coefficient is to be examined for significance.

Similar partitioning algorithm is applied to the other combined subbands as well as the composite coarsest subband.

$k=129, 513, 2049, 8193, 32{,}719$ are the leading indices from resolution level (*L* − 1) to level 1(finest resolution level). There is a total of five combined level of arrangement in eight MRI slices, where each of 128 × 128 resolution.

The 3D coefficients are mapped to a 1D array of length *I* after hybrid transformation. The progressive encoder encodes the most significant bit plane and moves towards the lowest bit plane. It can be stopped whenever the bit budget matches the target rate. The significance level for each bit plane is *s* = $2^n$, which is calculated with the bitwise logical AND operation $(\cap )$. The decoder performs reverse of encoding operation with some minor changes. The decoder generates the magnitude bits and sign bits of the coefficients with bitwise logical OR $(\cup )$ instead of bitwise logical AND $(\cap )$.

The 1D coefficient array $\xi$ is bit-plane coded and examined for significance in each bit plane pass. The initial threshold value can be computed as follows:

1 $$n = \left\lfloor {\rm log}_2 (\max_k \left| \xi (k) \right| ) \right\rfloor$$

1. The initialization of *Sm*[*k*] and *Dm*[*k*] state table markers are illustrated below:

- *Sm*[1, 17, 33, 49, 65, 81, 97, 113] = *Dm*[1, 17, 33, 49, 65, 81, 97, 113] = 3 for $LL_5$ subband.
- *Sm*[129, 177, 225, 273, 321, 369, 417, 465] = 
*Dm*[129, 177, 225, 273, 321, 369, 417, 465] = 4 are the leading nodes of $HL_5$, $LH_5$ and $HH_5$ subbands.
- *Sm*[513, 705, 897, 1089, 1281, 1473, 1665, 1857] = 
*Dm*[513, 705, 897, 1089, 1281, 1473, 1665, 1857] = 5 are the leading nodes of $HL_4$, $LH_4$ and $HH_4$ subbands.
$$\vdots$$
- *Sm*[2049, 2817, 3585, 4353, 5121, 5889, 6657, 7425] = 
*Dm*[2049, 2817, 3585, 4353, 5121, 5889, 6657, 7425]  = 6 are the leading nodes of $HL_3$, $LH_3$ and $HH_3$ subbands.
- *Sm*[8193, 11,265, 14,337, 17,409, 20,481, 23,553, 26,625, 29,697] = 
*Dm*[8193, 11,265, 14,337, 17,409, 20,481, 23,553, 26,625, 29,697]  = 7 are the leading nodes of $HL_2$, $LH_2$ and $HH_2$ subbands.
- *Sm*[32,769, 45,057, 57,345, 69,633, 81,921, 94,209, 106,497, 118,785] = 
*Dm*[32,769, 45,057, 57,345, 69,633, 81,921, 94,209, 106,497, 118,785]  = 8 are the leading nodes of $HL_1$, $LH_1$ and $HH_1$ subbands.

2. *Dm*[*k*] shall be initialize to an arbitrary value (i.e. $Dm[k]\ge (log_2N+1)+1$) and these are marked as INC.

### Block partitioning of 3D-HLCK algorithm

**Fig. 4:**
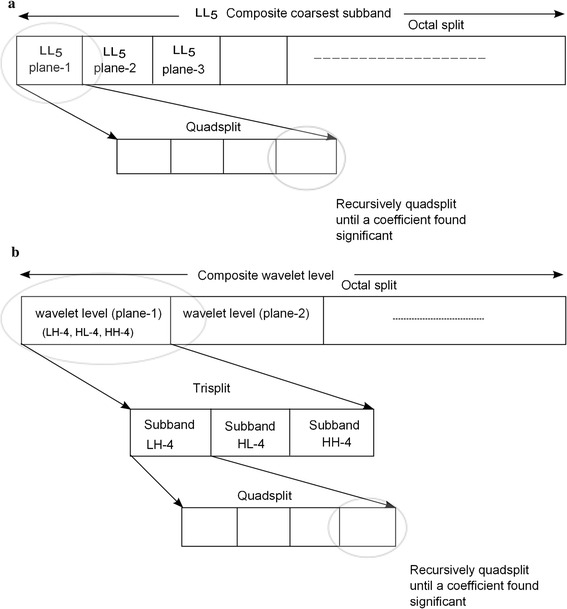
Partitioning of **a** combined coarsest subband, **b** combined wavelet level

The block partitioning of composite/combined levels is demonstrated in Fig. 4. Figure 4a demonstrates how the partitioning takes place for the composite coarsest level for the 1D arrangement of coefficients, and Fig. 4b demonstrates the partitioning of the combined pyramid level. If a coefficient is found to be significant, the combined coarsest level is partitioned into eight levels, where each level corresponds to the coarsest level of individual slices. Further, recursive quad partitioning in each level takes place until a coefficient is found to be significant. Finally, the significance of the coefficient value along with the sign bit will be transmitted. No sign bit will be transmitted if the coefficient is found to be insignificant. Similarly, the combined pyramid level is first octal partitioned into individual pyramid levels which correspond to each image slice. Then, each pyramid level is tri partitioned to find the subbands. The subbands are further quad partitioned to find a significant coefficient. Then, the coefficient will be coded and transmitted.

All the steps described above is presented below in the form of Pseudocode.

#### Pseudocode of 3D-HLCK algorithm

Bit plane pass1: Insignificant coefficient pass 
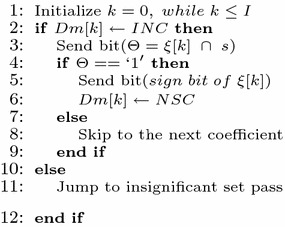


Bit plane pass2: Insignificant set pass
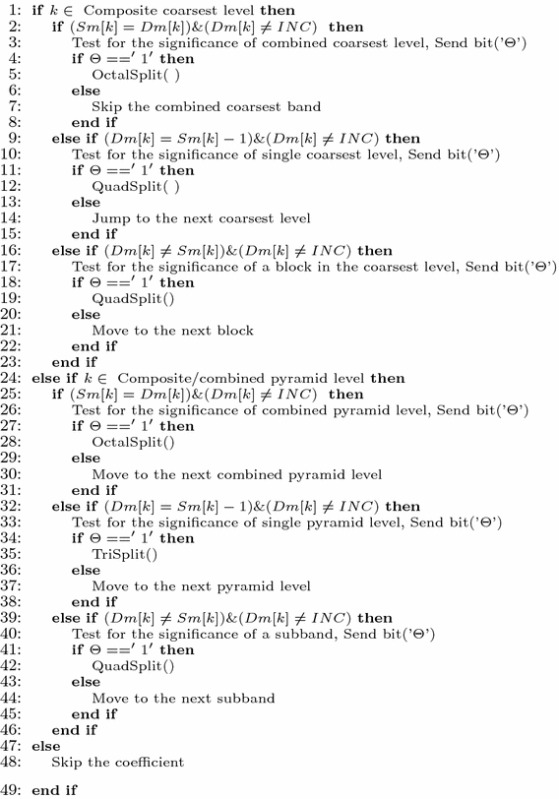


Bit plane Pass3: Refinement pass
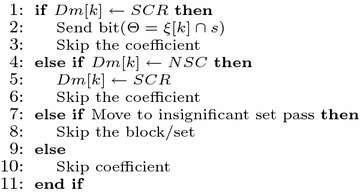


### Functions and parameters used in pseudocode

1. Significant test function $(\zeta _n(\gamma ))$: Significant test is obtained by logical AND $(\cap )$ operation.

#### *Example*

Let a (2 × 2) block $\gamma =[-127 \;109\; 19\; -24]$, and current threshold value $n=6$. $\zeta _{n}$ can be calculated as

2 $$\zeta_{\rm n} (\gamma ) =\sum \limits _{\rm all\, k} [(2^{\rm n} \le \left| \gamma ({\rm k}) \right| )\; \cap \;(\left| \gamma ({\rm k}) \right| \le {2}^{{{\rm n} + 1}} )]$$

$$\begin{array}{l} {\rm if} \;\zeta_{\rm n}(\gamma) = 0, {\rm then\; output} = 0 \\ {\rm else,\;partition\;the\;block}{.} \\ \end{array}$$

2. Function QuadSplit( ):

The function partition the subband into four equal block sizes. The algorithm for quard partitioning can be illustrated below:

$$\begin{array}{l} Dm[k] = Dm[k] - 1; \\ for\; j = 1,2,3 \\ \quad \quad Dm[k + (j \times 2^2 \times Dm[k]) ] = Dm[k] \\ end \\ \end{array}$$

Note that if $Dm[k]=0$, quad partitioning stops. The corresponding coefficient in block ‘$\gamma$’ is an insignificant coefficient (INC), and it will be examined for significance in insignificant coefficients pass (Pass 1) of the algorithm.

The OctalSplit() and TriSplit() functions are similar to the algorithm for QuadSplit(). OctalSplit() produces eight equal partitioned blocks, whereas TriSplit() produces three equal partitioned blocks.

If block ‘$\gamma$’ is a composite coarsest subband, then ‘$\gamma$’ undergoes octal partitioning (shown in Fig. 4a). Each partitioned block belongs to the coarsest wavelet level of the extracted plane of 3D medical image.

If block ‘$\gamma$’ is a composite/combined wavelet level, then ‘$\gamma$’ also undergoes octal partitioning (shown in Fig. 4b). Each partitioned corresponds to a wavelet level having three subbands.

3. If $Dm[k] = Sm[k]$, then a combined wavelet level is to be tested for significance.
4. If $Dm[k] = Sm[k]-1$, then a single wavelet level is to be tested for significance.
5. If $Dm[k] = Sm[k]-2$, then a subband is to be tested for significance.

### Comparison with listless embedded block partitioning (LEBP)

The main differences between our earlier work on LEBP algorithm (Senapati et al. 2014b) and 3D-HLCK are:

1. A 3D hybrid transform is used in 3D-HLCK (Wavelet transform using CDF 9/7 filters (Daubechics and Sweldens 1998) along spatial dimension and KLT along spectral dimension), whereas 2D wavelet transform is used in LEBP algorithm.
2. The 3D coefficient arrangement is mapped to an 1D arrangement in order to encode large clusters of insignificant coefficients in 3D-HLCK. However, LEBP uses 2D to 1D mapping scheme.
3. Rate reduction because of fixed state table (*Sm*[*k*] markers) at initial passes in 3D-HLCK. For example, $Sm[k]=Dm[k]$ indicates a composite wavelet level can be skipped instead of a single wavelet level as in LEBP.
4. Separate encoding techniques are used in 3D-HLCK for combined coarsest and combined wavelet levels so as to reduce the number of zeros for insignificant coefficients in the coarsest subband.

### Memory allocation

In 3D-HLCK, the mapped 3D coefficient array, $L_{max}$ has length 8*I*, where *I* is a 1D length of each slice/plane. If *Y* bytes are allocated for each subband coefficient, then the total storage memory required is 8*IY* for the subband coefficients and *RC* / 2 for the Dynamic state table $\mathbf{Dm}$ as each marker is half a byte. In the case of *L* level of wavelet decomposition, $\mathbf{Sm}$ needs $\frac{(8L+1)}{2}$ bytes, as the number of fixed markers are $(8L+1)$ and each marker is half a byte.

Hence, the total memory needed by 3D-HLCK is:

3 $$M_{3D-HLCK}=8IY+RC/2+(8L+1)/2.$$

As said earlier *Sm*[*k*] markers are fixed markers. These are used in association with *Dm*[*k*] markers to check for insignificance (refer to pseudocode).

In 3D-SPIHT coder, dynamic memory is determined by the auxiliary lists. The 3D-SPIHT uses of LIP, LIS, and LSP as auxiliary lists. LIS has type ‘A’ or ‘B’ information to distinguish the coefficients.

Let, $N_{LIP}$ be the number of coefficients in LIP, $N_{LSP}$ be the number of coefficients in LSP, $N_{LIS}$ be the number of coefficients in LIS, and *Y* be the number of bits to store the addressing information of a coefficient.

Then the total memory required (in bytes) due to auxiliary lists is given by Senapati et al. (2014a):

4 $$M_{3D-SPIHT}= [Y(N_{LIP}+N_{LIS}+N_{LSP})+ N_{LIS}]/8$$

As the memory size increases in each bit plane pass, The worst case values are,

5 $$N_{LIP}+N_{LSP}=3\times M \times N, N_{LIS}=3\times (M \times N)/4.$$

The memory required by Jyotheswar and Mahapatra (2007) is $\left(\frac{37}{16}+\frac{5}{16}\times (Y+1)\right)\times M\times N\times$ (No. of planes).

For a 128 × 128 image using 3 bytes per coefficient and five levels of wavelet transform, and having the optional pre-computed maximum length array (i.e, 8*IY* for 3D-HLCK), the worst case memory (RAM) required is $\frac{(128\times 128)}{2}\times (8\,{\rm bits})+\frac{(8L+1)}{2} \simeq 8$ kB for 3D-HLCK, 204 kB for 3D-SPIHT and 60 kB by Jyotheswar and Mahapatra (2007). Therefore 3D-HLCK is a suitable candidate over 3D-SPIHT and work in Jyotheswar and Mahapatra (2007) in terms of memory saving. This calculation is based using only memory consumption by the algorithms without regard to wavelet transform. Efficient wavelet transform techniques that take less memory have been reported recently in Mendlovic et al. (1997).

## Results and discussion

**Fig. 5:**
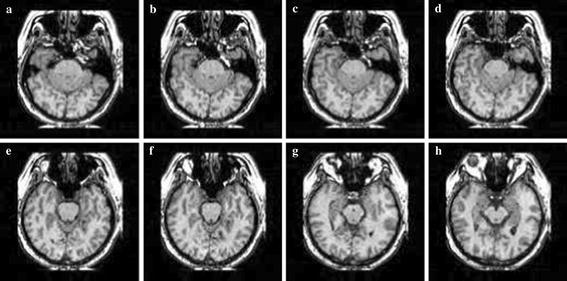
Compressed MRI image slices (**a**–**h**) by 3D-HLCK at a BR = 1.0 bpp

**Fig. 6:**
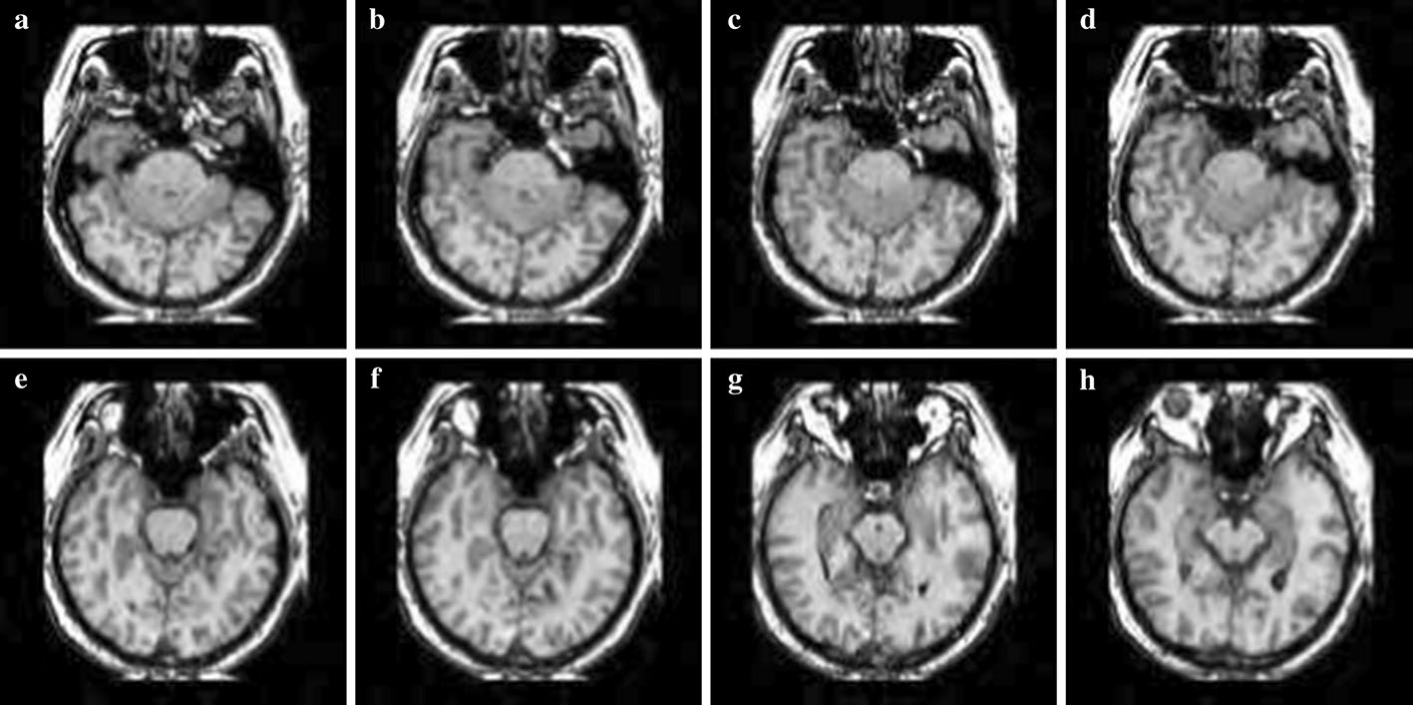
Compressed MRI image slices (**a**–**h**) by Jyotheswar and Mahapatra (2007) at a BR = 1.0 bpp

**Fig. 7:**
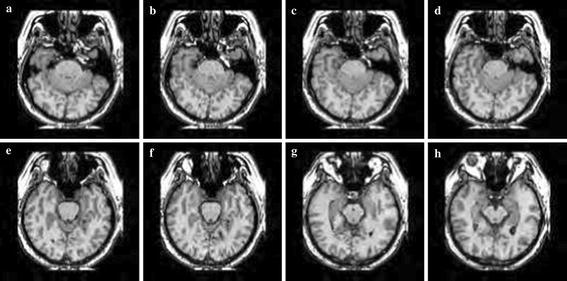
Compressed MRI image slices (**a**-**h**) by 3D-SPIHT at a BR = 1.0 bpp

**Fig. 8:**
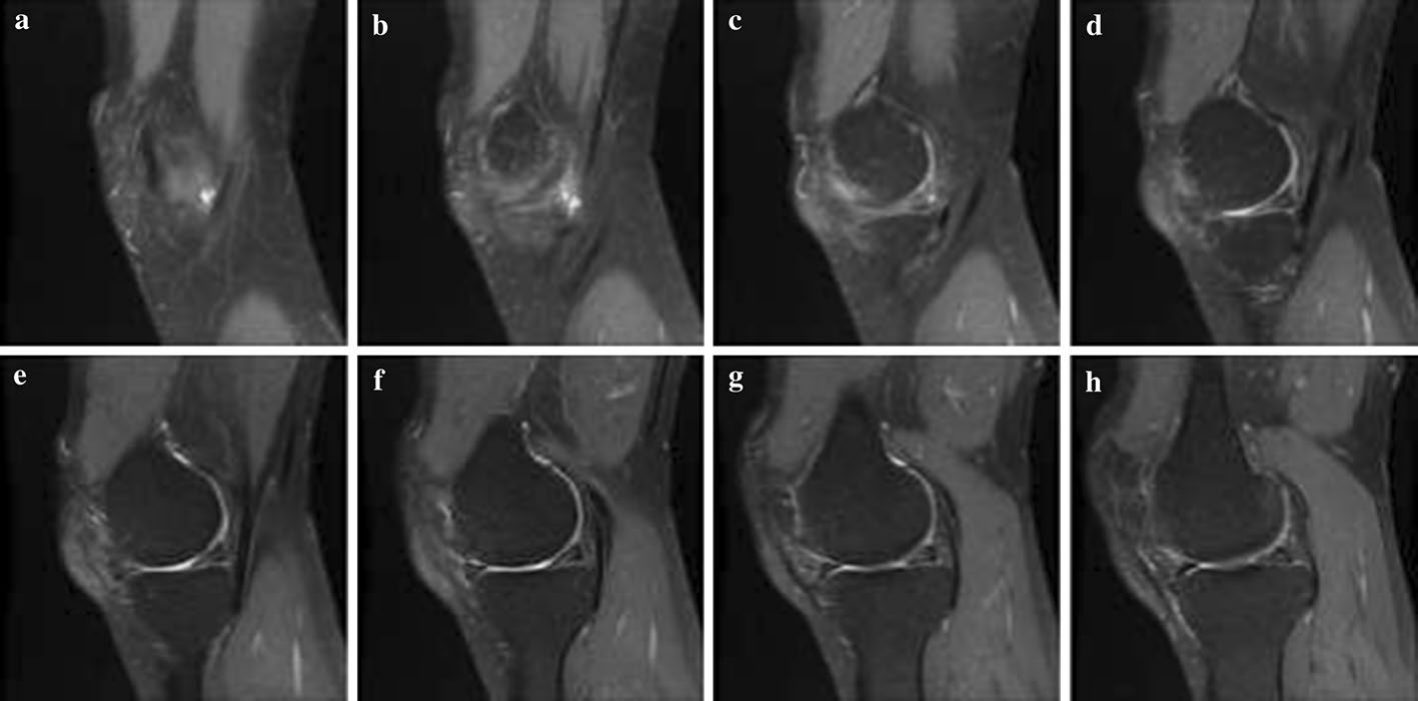
Compressed DICOM image slices (**a**–**h**)at a BR = 2.0 bpp

**Fig. 9:**
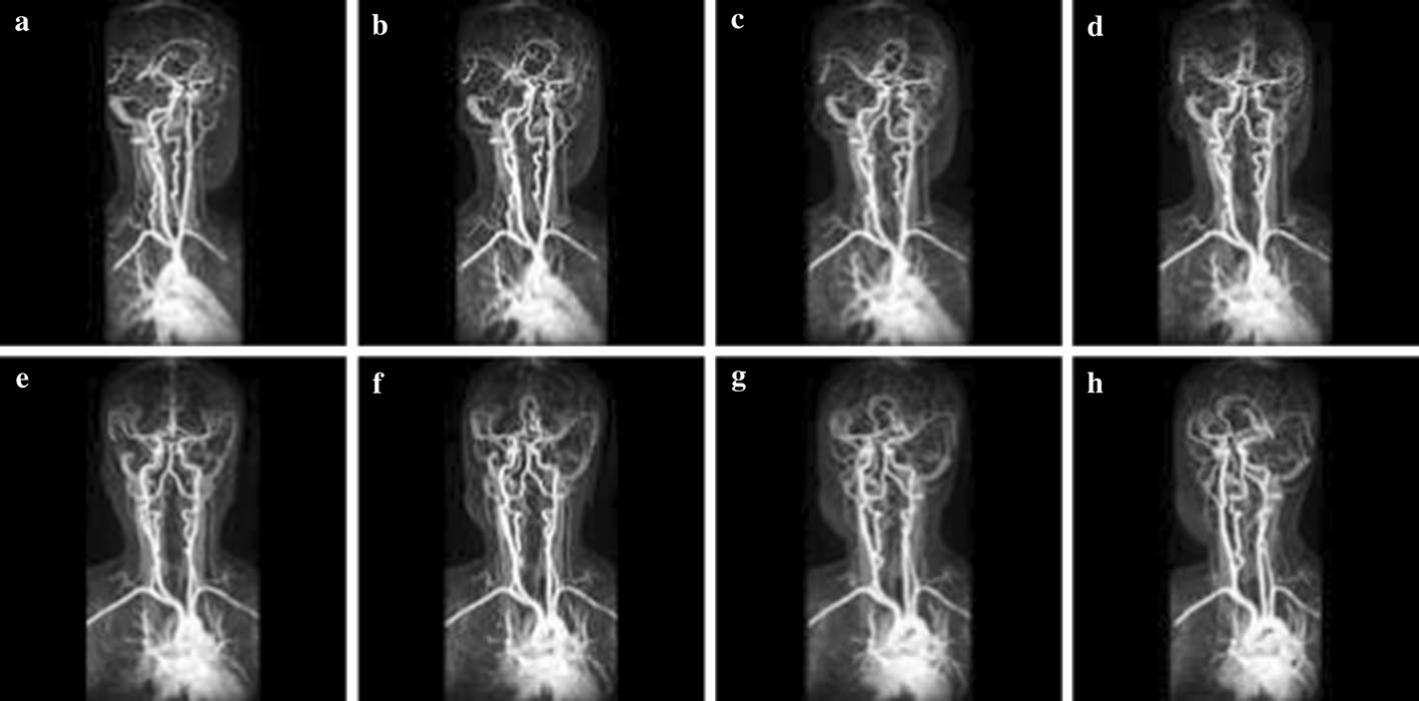
Compressed angiogram image slices (**a**–**h**) at a BR = 2.0 bpp using 3D-HLCK

Simulation was carried out on a Window XP platform having an Intel core i5 processor operating at a frequency of 2.6 GHz and 6 GB of internal RAM. The bit rate was varied from 0.5 to 2 bpp for compressing the images. Brain MRI, DICOM knee, and angiogram images were used in our experiment. Each image with a size 128 × 128 was used for the experiment. Tables 1, 2 and 3 summarise the PSNR comparison between 3D-SPIHT (Sriram and Shyamsunder 2011), the algorithm by Jyotheswar and Mahapatra (2007) and the proposed 3D-HLCK algorithm for brain MRI images. Tables 4, 5 and 6 summarise the PSNR comparison for DICOM knee images. Figures 5, 6, and 7 show compressed brain MRI images at a bit rate of 1.0 bpp using 3D-HLCK, the algorithm in Jyotheswar and Mahapatra (2007), and 3D-SPIHT respectively. It is apparent from the Figs. 5, 6, and 7 that the visual quality of the compressed images using 3D-HLCK is better than that obtained by using 3D-SPIHT and comparable with the algorithm in Jyotheswar and Mahapatra (2007). Figures 8 and 9 show the DICOM knee and angiogram images compressed at 2.0 bpp using proposed 3D-HLCK algorithm.

**Table 1:** PSNR comparison of brain MRI image at 0.5 bpp

Algorithm	Slice-1	Slice-2	Slice-3	Slice-4	Slice-5	Slice-6	Slice-7	Slice-8
3D-SPIHT (Sriram and Shyamsunder 2011)	24.7942	24.8550	25.2461	24.9937	25.2052	25.3051	24.7844	24.9987
Jyotheswar and Mahapatra (2007)	24.8210	24.9234	25.3012	25.1224	25.3045	25.3412	24.7724	25.0654
3D-HLCK	25.0136	25.4772	25.6954	25.1605	25.3025	25.5581	24.8334	25.0457

**Table 2:** PSNR comparison of brain MRI image at 1.0 bpp

Algorithm	Slice-1	Slice-2	Slice-3	Slice-4	Slice-5	Slice-6	Slice-7	Slice-8
3D-SPIHT (Sriram and Shyamsunder 2011)	28.7479	29.3962	29.6978	28.8805	28.7790	28.9684	28.6158	29.3115
Jyotheswar and Mahapatra (2007)	29.1123	29.9129	29.8125	29.2224	29.4042	29.5512	28.9724	29.4654
3D-HLCK	28.9032	29.7086	29.7709	29.0900	29.3314	29.4190	28.8109	29.3783

**Table 3:** PSNR comparison of brain MRI image at 2.0 bpp

Algorithm	Slice-1	Slice-2	Slice-3	Slice-4	Slice-5	Slice-6	Slice-7	Slice-8
3D-SPIHT (Sriram and Shyamsunder 2011)	35.5649	35.8673	35.7901	35.5476	35.5843	35.6065	35.7257	35.6406
Jyotheswar and Mahapatra (2007)	35.7210	35.9524	35.9612	35.8128	35.9245	35.8322	35.9724	35.9654
3D-HLCK	35.6260	35.9328	35.9546	35.7196	35.9179	35.7822	35.8127	35.8426

**Table 4:** PSNR comparison of DICOM knee image at 0.5 bpp

Algorithm	Slice-1	Slice-2	Slice-3	Slice-4	Slice-5	Slice-6	Slice-7	Slice-8
3D-SPIHT (Sriram and Shyamsunder 2011)	35.6328	35.2043	34.2868	34.9760	34.9612	34.9178	34.6008	34.3115
Jyotheswar and Mahapatra (2007)	35.8276	35.3610	34.9234	35.3578	35.2997	35.2491	34.7612	34.4321
3D-HLCK	35.8646	35.3801	34.9428	35.3693	35.3564	35.2795	34.8423	34.4471

**Table 5:** PSNR comparison of DICOM knee image at 1.0 bpp

Algorithm	Slice-1	Slice-2	Slice-3	Slice-4	Slice-5	Slice-6	Slice-7	Slice-8
3D-SPIHT (Sriram and Shyamsunder 2011)	38.9826	38.7107	38.4028	38.5471	38.7088	38.5060	38.3513	37.8718
Jyotheswar and Mahapatra (2007)	39.0214	38.8820	38.5221	38.7510	38.8997	38.6126	38.3901	37.9011
3D-HLCK	39.1471	38.8796	38.5239	38.7847	38.9222	38.6316	38.4140	37.9713

**Table 6:** PSNR comparison of DICOM knee image at 2.0 bpp

Algorithm	Slice-1	Slice-2	Slice-3	Slice-4	Slice-5	Slice-6	Slice-7	Slice-8
3D-SPIHT (Sriram and Shyamsunder 2011)	44.3862	44.2377	43.5063	43.9376	43.9620	43.7279	43.7797	43.4163
Jyotheswar and Mahapatra (2007)	44.3901	44.3213	43.9112	44.2011	44.1930	43.9902	43.9128	43.5234
3D-HLCK	44.3887	44.3083	43.8962	44.1202	44.1539	43.9502	43.8313	43.5019

### Discussion

Comparisons of the coding performance (PSNR vs. Slice no.) for MRI brain image, DICOM knee image, and MRI angiogram images for a constant bit rate are summarised in Tables 1, 2, 3, 4, 5, 6, 7 and 8 respectively. It is observed that the proposed 3D-HLCK algorithm exhibits a PSNR improvement between 0.05 and 0.5 dB for MRI brain images and 0.05–0.6 dB for DICOM knee images for a bit rate of 0.5–2.0 bpp compared to 3D-SPIHT. 3D-SPIHT shows higher a PSNR in the Slice-1, Slice-2, and Slice-7 images in comparison to 3D-HLCK in the MRI angiogram images at 1 bpp. A similar trend is also observed at 2 bpp. In comparison to the work by Jyotheswar and Mahapatra (2007), 3D-HLCK shows an improvement of 0.05–0.5 dB for the given slices at 0.5 bpp. However, at higher rates, the work in Jyotheswar and Mahapatra (2007) shows a PSNR improvement of around 0.15 dB in the MRI images compared to 3D-HLCK. In the DICOM and angiogram images, the algorithm in Jyotheswar and Mahapatra (2007) shows a slight PSNR improvement with respect to 3D-HLCK.

**Table 7:** PSNR comparison of MRI angiogram image at 1.0 bpp

Algorithm	Slice-1	Slice-2	Slice-3	Slice-4	Slice-5	Slice-6	Slice-7	Slice-8
3D-SPIHT (Sriram and Shyamsunder 2011)	36.3810	36.5521	36.0900	36.4401	36.4912	36.3016	37.0721	36.5801
Jyotheswar and Mahapatra (2007)	36.4891	36.4389	36.2523	36.5013	36.4434	36.4012	37.0121	36.6010
3D-HLCK	36.2908	36.4508	36.2815	36.5191	36.4380	36.3937	36.9916	36.0091

**Table 8:** PSNR comparison of MRI angiogram image at 2.0 bpp

Algorithm	Slice-1	Slice-2	Slice-3	Slice-4	Slice-5	Slice-6	Slice-7	Slice-8
3D-SPIHT (Sriram and Shyamsunder 2011)	45.1229	45.2415	45.0016	44.7210	44.6910	44.7890	44.8010	44.7892
Jyotheswar and Mahapatra (2007)	45.1321	45.2312	45.1200	44.8310	44.7610	44.8231	44.8012	44.7891
3D-HLCK	44.8976	45.1531	45.1106	44.8234	44.7794	44.8434	44.8144	44.8627

The proposed algorithm exhibits a better PSNR improvement for other slices in 3D-SPIHT because of the following reasons:

1. 3D SPIHT uses 3D DWT coefficients for encoding, whereas hybrid transformed (2D DWT+KLT) coefficients are encoded by 3D-HLCK.
2. Large clusters of zeros are efficiently coded (both inter and intra) by 3D-HLCK.
3. Coefficients are efficiently arranged among different subbands of slices to exploit inter- and intra-subband correlations within and across slices.

The work in Jyotheswar and Mahapatra (2007) outperforms 3D-HLCK at higher rates (above 1 bpp) for MRI images for the following reasons: (i) The execution of a refinement pass before the sorting pass. (ii) The ordering of the coefficient scanning process for simple hardware implementation. (iii) Optimisation for lossless encoding using 5/3 filters in the spatial and spectral dimensions.

The proposed 3D-HLCK algorithm will occupy a fixed amount of memory, irrespective of the number of bit-plane passes, owing to the fixed number of state table markers. Partitioning takes place by updating the marker values. Each marker holds a maximum 4 bits. The algorithm in Jyotheswar and Mahapatra (2007) requires a fixed memory size and exhibits simple hardware portability. However, in 3D-SPIHT, the linked lists (LIP, LIS, and LSP) add/remove/move additional nodes for every bit-plane pass. Therefore, the memory usage grows exponentially. Rate and resolution scalability on par with 3D-SPIHT is achieved by 3D-HLCK. Memory saving is trivial, as in most applications, the cost of memory is cheap. However, the proposed algorithm is potentially suitable for applications such as the progressive transmission of DICOM images, lossless archival, telemedicine, teleradiology, and capsule endoscopy. Therefore, 3D-HLCK can be a preferred option over 3D-SPIHT for the aforementioned applications. A further reduction in the overall complexity can be achieved by using fractional wavelet transforms (FrWTs) (Mendlovic et al. 1997) for such applications.

From the simulation, it is observed that the average encoding and decoding times for 3D-HLCK are 12 times more than those for 3D-SPIHT at 2 bpp. Further optimisation can be done for 3D-HLCK to reduce the time complexity. However, it can be proved mathematically that the computational complexity of 3D-HLCK will be O(N) operations compared to O(N log N) for 3D-SPIHT (Senapati et al. 2014a).

## Conclusion

A new 3D coder called 3D-HLCK is proposed in this paper. Owing to the listless nature of 3D-HLCK, significant memory reductions of over 96 and 86% are achieved compared to 3D-SPIHT and the work by Jyotheswar and Mahapatra respectively. 3D-HLCK has features such as rate and resolution scalability. In brain MRI, DICOM knee and angiogram images, a PSNR improvement of 0.05–0.5 dB is also achieved compared to 3D-SPIHT. The proposed coder exhibits a comparable coding efficiency and easy hardware portability with the work by Jyotheswar and Mahapatra. Therefore, it can be used in applications such as telemedicine, teleradiology, wireless capsule endoscopy and the Internet transmission of DICOM images. Future work will incorporate additional features such as the ROI coding, random access coding, and video coding using 3D-HLCK.
